# In Search of an Integrative Measure of Functioning

**DOI:** 10.3390/ijerph120605815

**Published:** 2015-05-26

**Authors:** Rosamond H. Madden, Nick Glozier, Nicola Fortune, Maree Dyson, John Gilroy, Anita Bundy, Gwynnyth Llewellyn, Luis Salvador-Carulla, Sue Lukersmith, Elias Mpofu, Richard Madden

**Affiliations:** 1 Centre for Disability Research and Policy, University of Sydney, P.O. Box 170, NSW 1826, Australia; E-Mails: nicola.fortune@sydney.edu.au (N.F.); john.gilroy@sydney.edu.au (J.G.); gwynnyth.llewellyn@sydney.edu.au (G.L.); luis.salvador-carulla@sydney.edu.au (L.S.-C.); richard.madden@sydney.edu.au (R.M.); 2 Brain & Mind Research Institute, Sydney Medical School, University of Sydney, 94 Mallett St., Camperdown, NSW 2050, Australia; E-Mail: nick.glozier@sydney.edu.au; 3 National Centre for Classification in Health, University of Sydney, P.O. Box 170, NSW 1826, Australia; 4 Dyson Consulting Group, 450 Chapel Street, South Yarra, VIC 3141, Australia; E-Mail: maree@dysonconsultinggroup.com.au; 5 Faculty of Health Sciences, University of Sydney, East Street, Lidcombe 2141, Australia; E-Mails: anita.bundy@sydney.edu.au (A.B.); elias.mpofu@sydney.edu.au (E.M.)

**Keywords:** functioning, disability, rehabilitation, public health, people-centred services, integrated care, measurement, assessment, ICF

## Abstract

International trends towards people-centred, integrative care and support require any measurement of functioning and disability to meet multiple aims. The information requirements of two major Australian programs for disability and rehabilitation are outlined, and the findings of two searches for suitable measures of functioning and disability are analysed. Over 30 current measures of functioning were evaluated in each search. Neither search found a generic measure of functioning suitable for these multibillion dollar programs, relevant to a wide range of people with a variety of health conditions and functioning experiences, and capable of indicating support needs, associated costs, progress and outcomes. This unsuccessful outcome has implications internationally for policy-relevant information for disability, rehabilitation and related programs. The paper outlines the features of an Integrative Measure of Functioning (IMF) based on the concepts of functioning and environmental factors in the International Classification of Functioning, Disability and Health (ICF). An IMF would be applicable across a variety of health conditions, settings and purposes, ranging from individual assessment to public health. An IMF could deliver person-centred, policy-relevant information for a range of programs, promoting harmonised language and measurement and supporting international trends in human services and public health.

## 1. Introduction

Measurement of functioning and disability in the 21st century increasingly takes place in the context of complex relationships and interactions among people, communities, services and systems. One result of this complexity has been the development of a growing array of specialised measurement instruments, specific to purpose, health condition, setting or service provider. An alternative approach, particularly relevant for large national programs, is to seek or to develop an integrative, generic measure, relevant to diverse purposes and populations.

This paper examines Australian experience with two significant national programs and their unsuccessful search for a suitable measure of functioning. It goes on to set out the case for developing a generic, integrative measure of functioning (IMF), for use in rehabilitation, disability support, and related fields. Here we use “generic” to mean that such a measure would be applicable across conditions, settings and purposes, and “integrative” to mean that it would support integrated human services systems focussed on the individual and providing a continuum of care and would use a common language focussed on people’s needs, outcomes and environments.

Designing measurement tools suited to broad national programs requires the specification of policy purposes, of information requirements within the policy framework, and of measurement concepts relevant to the information required. A design process, often iterative, is needed to ensure that (a) the measurement concepts, as operationalised in the measurement instrument, provide the information sought; and (b) the information produced serves the identified policy purposes [1,2] (p. 187).

Measurement must not only be fit for purpose, but also “fit for process”. That is, measurement processes should contribute to and fit in with the day-to-day procedures of service provision and administration and produce value for the people who are involved in providing and recording the information [3]. For person-centred, services the person is integral to goal setting and measurement, and measurement must genuinely involve the person [1,4,5]. 

Designing measurement tools is assisted by the use of standard frameworks and concepts. The International Classification of Functioning, Disability and Health (ICF) [6] is a world standard framework and classification, adopted by the World Health Assembly for use in all nations. Its model depicts functioning and disability as multidimensional—described in terms of body functions and structures, activities and participation—and influenced by a person’s environment as well as their health conditions. It is consistent with rights-based policies embodied in and derived from the United Nations Convention on the Rights of Persons with Disabilities 2006 [7,8]. The nine Activities and Participation chapters of the ICF are: Learning and applying knowledge; General tasks and demands; Communication; Mobility; Self care; Domestic life; Interpersonal interactions and relationships; Major life areas (including education, work and employment, and economic life); and Community, social and civic life. The five chapters listing Environmental Factors (which can be either facilitators or barriers) are: Products and technology; Natural environment and human-made changes to environment; Support and relationships; Attitudes; Services, systems and policies.

Health and human service systems are increasingly focused on person-centred approaches, capable of supporting people over time and across different system components [5,9]. The individual’s “functioning” is a key component of health and well-being and requires direct consideration in these systems. The necessary interconnectedness of these systems requires a common language about functioning for communication among the people, providers and systems concerned, and a common framework to underpin measurement and information about functioning [5,10,11]. The ICF provides this common language and framework, as well as the detailed building blocks for measurement and information about functioning and health [6,12]. 

The use of the ICF facilitates the selection of “what” to measure or assess once we know the purpose of measurement. Based on more than a decade of use, there is a growing body of knowledge about its use [1,13]. In specific populations, sub-sets of ICF domains may be selected for use. For example, several “core sets” of ICF categories, relevant to particular health conditions, have been developed to facilitate the use of the ICF in clinical practice, particularly in rehabilitation [14]. There is also published advice, referring to the ICF, to clinicians on “appropriate attributes and standards required for assessment and outcome measurement” [15].

The ICF has also been used as a framework for more general measures. The World Health Organization Disability Assessment Schedule, the WHODAS 2.0, is a survey instrument developed by the World Health Organization (WHO), based on the ICF and covering most of the ICF Activities and Participation chapters [16]. In Australia, the ICF is used in national data standards and in statistical collections [17]. 

As well as being used as a basis for developing new instruments, the ICF has been used extensively to link existing instruments [18]. The ICF was found to be useful for classifying the content of the instrument development platform and databank PROMIS [19]. It has been proposed that, by applying linking rules and Rasch methods, the ICF can be used as a “unified framework” for reporting functioning, irrespective of the original instrument used for measurement [20]. 

Despite these activities, there has been no development of a new generic measure designed to be suitable for diverse populations, for assessing support needs for and monitoring of large national programs in the health and disability fields.

Appropriate and ethical design and application of measurement tools are essential foundations for assessment processes that deliver benefit to the person whose functioning is being assessed. Accordingly, the ICF provides ethical guidelines for its use [6,13]. Evaluation of the benefits and costs to all stakeholders must also form part of developing and testing an instrument. A related important and practical consideration is balancing parsimony with reduction in measurement error (*i.e.*, not wasting people’s time for no gain in precision).

In the body of literature that has grown up around the application of the ICF to the measurement of functioning, three particular challenges have been noted: how to build in a consideration of the environment and its effect on a person’s functioning (as recognised in the ICF); how to set “thresholds”, where required, to delineate groups of people with different levels of functioning (relevant to both population prevalence estimates and to assessment of eligibility for defined programs); and how to combine the perspective of the person concerned with the perspectives of various providers, in relation to the person’s functioning [1].

## 2. Method: Analysis of Two Australian Searches for Measurement Tools

Two major national programs in Australia searched unsuccessfully for a suitable, generic measure of functioning, relevant to diverse populations and adaptable for use across the life span [21,22]. This section provides a brief outline of the programs, the related information needs, and the searches (a number of the co-authors of this paper were involved in these searches).

### 2.1. A National Program Funding Disability Supports

Australia’s National Disability Insurance Scheme (NDIS) aims to “support the independence and social and economic participation of people with disability”, provide funding for “reasonable and necessary supports”, and “enable people with disability to exercise choice and control in the pursuit of their goals and the planning and delivery of their supports” (NDIS Act ss.3, 34, 35). The NDIS Act (s.3) situates the NDIS as one of the policy instruments that gives effect to Australia’s obligations under the UN Convention on the Rights of Persons with Disabilities. To become a participant of the Scheme it must be established that, among other requirements, a person has an impairment that “results” in substantially reduced functional capacity in activities and participation; people may also qualify if the provision of early intervention supports is likely to reduce the person’s future needs (NDIS Act ss.24, 25). Participants are provided with individualised funding with which to purchase the supports identified (NDIS Act ss.42, 43). Implementation of the Scheme began in July 2013, in trial sites across Australia. The ICF was recommended for use in the development and implementation of the Scheme [23].

It was recognised during the planning for establishing NDIS pilot sites in July 2013 that administration of the Scheme requires recording and measurement instrument(s) able to provide information and data that can be used to: establish the presence of an impairment, activity limitation or participation restriction; inform the determination of the extent of support needed for activities and participation, in line with both the person’s goals and what might be considered “reasonable and necessary”; and monitor progress over time. To inform eligibility for the scheme, a measure would need to be capable of demonstrating “reduced functional capacity to undertake, or psychosocial functioning in undertaking” activities, and reduced “capacity for social and economic participation” (NDIS Act, s.24). The measure should also produce information on need for assistance that can be used both to inform decisions about the package of supports needed for an individual and to predict costs of funding supports for the scheme more broadly. The person’s goals and “supports” needed could relate to any of the 5 domains of environmental factors listed in the ICF—e.g., support in the form of equipment, personal assistance, building modifications, professional support, improved access to health care or other services, or efforts to improve community knowledge and attitudes.

The measure should enable the evaluation of progress and outcomes, both for individuals and across the Scheme. Change in the level of support needed could provide a key outcome indicator for both the person and the NDIS. To cover the full range of participation goals (and related outcomes) that an NDIS participant may have, and life areas where they need supports, the tool should span the full range of ICF Activities and Participation domains. 

During the planning for the NDIS implementation, some 30 instruments were reviewed to identify any that could be used to assess the support needs of eligible participants and inform decisions on resource allocation [21]. This process identified some instruments that were widely accepted and relevant for use with children [24,25] or adults with psychiatric disability [26,27,28]. However, no instruments relevant for use with adults across a broad range of disabilities were found (Supplementary Table S1). Instruments were judged unsuitable for a variety of reasons, e.g., they did not include all critical ICF domains, were too long, or their validity across a broad range of disabilities was not established. No instruments were found that spanned the support needs of people regardless of the related health condition.

### 2.2. Funding of Sub-Acute Care

The Australian National Health Reform Agreement established activity based funding as the mechanism for funding public hospital services in Australia [29]. Under activity based funding, hospitals were to be funded on the basis of activity, by applying a “national efficient price” that is determined for each category of service. 

Activity based funding for sub-acute services, notably rehabilitation, commenced in July 2013. For sub-acute care, functioning information is particularly important as both a determinant of care planning and a predictor of the cost of care. Existing casemix classification systems for sub-acute care use the scores on specified functional assessment instruments (FIM, RUG-ADL, and HoNOS; see e.g., [30,31,32]) to group episodes of care. These groupings are used as a basis for funding service episodes. Following initial work, which had shown limitations of these instruments for this purpose [33], the National Centre for Classification in Health was commissioned by the Independent Hospital Pricing Authority to review existing assessment instruments and identify tools for inclusion in the casemix classification system to improve its ability to account for variance in resource intensity and length of stay [22]. The review included a stakeholder survey and consultation, and mapping of 33 existing instruments to the ICF (Supplementary Table S2).

In discussing the results of the review there was acknowledgement of the difficulty of balancing the competing demands of instrument sensitivity, an absence of ceiling and floor effects, clinical utility, ease of completion, and the need for the instrument to be usable across settings. The FIM, which is used in the current casemix classification for grouping episodes of rehabilitation care, was generally well accepted for inpatient rehabilitation, but not necessarily for other settings. Some stakeholders criticised it because of ceiling effects and the inadequacy of its cognition domains [34]. FIM ceiling effects were also reported in the literature reviewed, in relation to conditions such as brain injury, spinal cord injury, multiple sclerosis and stroke [35,36,37,38].

A key message from stakeholder consultations was that rehabilitation care provided to the full spectrum of patients in both admitted and non-admitted settings requires consideration of the full range of ICF Activities and Participation domains [22] (p. 58). The omission of key Activities and Participation domains from the FIM was of concern to some clinicians dealing with people with complex rehabilitation needs. There was broad agreement on the need for expansion of the range of domains in the FIM to describe need for assistance in non-admitted rehabilitation programs.

A number of criteria were identified as a basis for assessing the instruments: an instrument should cover the full range of ICF Activities and Participation domains, measure need for assistance, be well validated, and be easily completed by staff. It should be capable of predicting expenditure. The instrument should also provide clinically meaningful and credible information on functioning across different settings (inpatient and non-admitted). 

None of the instruments reviewed met all the criteria. To meet the information needs for activity based funding of sub-acute services in the medium term, the report recommended using a combination of existing instruments, to provide better coverage of all relevant domains. In the longer term, the development of a new tool was proposed [22]. 

A prototype instrument for use in rehabilitation, the AusRehab, was outlined to illustrate the potential for developing an ICF-based tool that met all the criteria and could be used to group episodes of care. The instrument covered all 9 ICF Activities and Participation chapters using a set of 18 items, selected with reference to the WHODAS 2.0 model, and with a five point scale to describe different levels of need in a way that relates to cost. (WHODAS 2.0 itself was not useable as it did not meet all criteria, in particular it did not measure need for assistance.)

## 3. Results 

The analysis of these two searches in Australia, for instruments for the national disability support program and for the national activity-based hospital funding system for rehabilitation and other sub-acute care, reveals common challenges and points to apparently similar solutions. For both programs what was being sought was a generic tool, relevant to a wide range of people, with a wide variety of health conditions and functioning experience or “status”. For the NDIS the primary purpose was indicating disability support needs and associated costs, as well as progress and outcomes; for activity-based funding of sub-acute care, the purpose was enabling pricing of sub-acute care, including rehabilitation. In both fields it was concluded that the desired tool should be ICF based and cover the full range of Activities and Participation chapters, and that the primary relevant measurement concept was “support” or “assistance with functioning”. Because of the breadth of the programs and the diversity of the populations served, instruments that were specific to health conditions or settings could not be used in either national program.

These findings demonstrate that there is a need for a generic, integrative measure of functioning (IMF), applicable in rehabilitation, disability support, and related fields. It must be ICF based, and must take into account all areas of life represented in the ICF Activities and Participation domains, as well as the environmental factors that affect them.

## 4. Discussion

### 4.1. Towards an Integrative and Generic Measure of Functioning, Disability and Health

How internationally relevant might these findings be, namely that two large national programs with different purposes require common measurement solutions? The instruments in use in Australia at present, including those examined in these two national searches, are widely used in other countries, and their shortcomings are not unique to Australia. Similarly, programs providing supports to people with disability exist in many countries; the UN Convention on the Rights of Persons with Disabilities recognises the need for a twin track approach to disability—access to specialist support services (such as the NDIS in Australia) as well as to mainstream services (such as rehabilitation). The problems of rehabilitation funding are equally global. There is interest in a number of countries in broadening hospital casemix funding methods to include functioning measures in addition to diagnoses and interventions [39,40,41]. Thus the findings of these two Australian searches—and their similarity—could be expected to be of interest in policy development and information management in other countries.

### 4.2. The Reasons to Consider a New Integrative Measure of Functioning

There are at least four related reasons for considering the development of a new integrative measurement of functioning (IMF).

#### 4.2.1. The Modern Policy and Service Context Requires Integrative Measurement

First, there is the evolving policy and service context across human services systems. The international trend is towards “integrative” and “person-centred” policies and services in the health and disability fields [9,42,43]. There is recognition of the potential for service integration to improve cost effectiveness, accessibility and quality of services. Improved integration requires shared recording practices and data, and comparative analysis of resulting data [42]. 

Modern health systems recognise the importance of enhancing human functioning in addition to diagnosis and disease prevention. “Functioning” is important in the context of chronic disease, mental health, and healthy development and ageing, as well as the rights of people with disabilities and their carers to participate in society [5]. The interfaces between “mainstream” services, such as health and education, and specialised disability-focussed services require attention to ensure a person-centred, rather than discipline-specific approach to functioning [4,44]. A person-centred approach requires integration across all relevant services (both specialist and mainstream), which in turn creates a need for measurement tools that can be used across services and sectors. 

Measurement frameworks and tools are needed which are relevant across purposes, programs, services, settings and time. A consistent approach to the measurement of functioning would bring with it the possibility of gaining a clearer understanding of how different programs relate to each other in terms of the people served and how the needs of individuals may or may not be met in different parts of the service system. 

#### 4.2.2. The Burden of Repeated Measurement of People’s Situation

Second, repeated assessment by service providers, using multiple measures across different but related programs and across a variety of settings, can be a burden for the person involved. The use of different concepts and language about functioning means that people are required to explain their situation repeatedly, using service-specific language, rather than language which relates directly to their functioning needs [5]. The burden of repeated measurement also results in costs to the overall system due to unnecessary duplication.

#### 4.2.3. The Risk of Using Inappropriate Instruments: the Example of Cost Prediction

Third, payers, be they public or private, wish to compare program metrics such as effectiveness and accessibility to assess allocative and productive efficiency, for instance by using measures of functioning to create profiles of service users that can be related to costs, or to predict which people will require high cost (or low cost) services and when. The current lack of suitable generic instruments, demonstrated by the analysis in previous sections, creates a risk that existing measurement tools are used in new applications without careful evaluation of their “fitness for purpose”. The use of unsuitable instruments can skew the intentions of the program—for instance, if key domains of functioning are not assessed, needs may not be met, and participation outcomes not achieved.

#### 4.2.4. The Need to Modernise and Integrate the Measurement of Functioning across Health and Disability Programs

Finally, there is a need to update existing measures to align with modern approaches to conceptualising functioning and disability [4,44] and to take advantage of the ICF’s potential as a common language and as a basis for unifying measurement across human services systems. There is widespread acceptance of the value of the ICF as a unifying framework and language and as a classification, as well as literature recognising its relevance to a range of fields. To date, however developments have tended to concentrate on specific health conditions and clinical applications [45,46]. 

For many people with functioning limitations, multi-morbidity is the norm. Measures focused on specific diseases or impairments fail to capture this adequately. The ICF is aetiologically neutral and can apply with any health condition or group of health conditions. Thus it provides the ideal building blocks for a comprehensive and setting-neutral generic measure of functioning. 

Measures of functioning across the health and disability fields require consideration of environmental factors [6]. Few existing measures embrace both functioning and the environmental factors that are recognised to be crucial to an adequate description and understanding of a person’s functioning [47]. The ICF contributes a neutral framework capable of recognising environmental factors as both facilitators and potential barriers (e.g., water, air, relationships, policies and programs); the field of public health brings a broad understanding of environmental factors already evidenced to be risk factors for disease or facilitators of health and functioning. Public health, with its broad policy focus on community and environment, is an obvious and useful bridge for harmonising the representation of environment across health and disability fields.

### 4.3. What are the Main Design Considerations for an Integrative Measure of Functioning?

The design of measurement tools—for national programs or for other broad purposes in diverse populations—requires the framing of policy and service purposes, of information requirements serving these purposes, and of measurement concepts relevant to the information required. We return to this theme from our introduction. The process of such framing requires collaborative development, involving the multiple stakeholders affected by measurement or using data derived from measurement. This framing entails work at different “levels” of these programs or systems (typically micro, meso and macro). In this paper we outline the criteria for a satisfactory IMF and indicate the collaborative methods required to develop and test it. While it is possible that the IMF could follow a familiar “matrix” pattern (a list of functioning domains intersected by a list of ratings), the discussion in this section outlines challenges and criteria that could take the IMF development in new directions.

#### 4.3.1. Purposes and Information Requirements for Integrative and Generic Measurement Tools

It has been suggested that the purposes of measurement and the related analyses can be grouped into three broad categories [1]: to identify areas of concern, and the support needed or given—at the individual or group level (for example, the NDIS information needs relate to this category, and to environmental interventions needed); to describe or compare functioning and disability—over time, or across settings or treatments (this is relevant to rehabilitation and other sub-acute care funding information needs, and to monitoring outcomes over time in the NDIS); to describe population health and well-being, and related environmental and other factors (this is of particular relevance in the field of public health; its focus on diversity requires the monitoring of outcomes for people with disabilities).

Once purposes are agreed on, broad information requirements can be identified and then transformed to detailed measurement elements and parameters.

#### 4.3.2. Domain Selection

In developing an Integrative Measure of Functioning (IMF), all components of disability described in the ICF (impairments, activity limitations, and participation restrictions) should be considered, as well as the environmental factors that affect them—even if it is decided, with sound reasons, not to apply them all. The interactions among components may themselves be of interest, for instance to understand the relationships between participation and environmental factors, or health conditions and disability overall [6]. All nine chapters of Activities and Participation are required to describe functioning in diverse populations (that is, populations with a varied range of people, health conditions and disabilities). It has been established that a subset of these domains cannot predict the whole picture of a person’s activities and participation in diverse populations [48]. 

Domain selection for an IMF would focus (at least initially) on the ICF Activities and Participation component (as explained in Section 2 and Section 3 above). There are pointers from previous research that may help in the selection of a parsimonious but meaningful set of domains which nevertheless cover all nine Activities and Participation chapters. Kostanjsek and colleagues propose a list of ICF categories to describe the “impact of health conditions generically across health conditions”, based on domains used in the WHODAS 2.0, in the World Health Survey, and in proposals for a “generic ICF core set” [12,16,49,50]. Kostanjsek’s work provides a key resource for consideration in building and testing an IMF. Determining the level of detail at which the domains should be represented involves balancing the richness of information captured against the time taken to complete the assessment.

#### 4.3.3. Measurement Constructs

Measurement constructs must be designed to be policy- and service-relevant—suited to the measurement purpose and information requirements.

The dominant measurement constructs used in current instruments are “difficulty” with functioning and “assistance needed” with functioning. The review of instruments for activity based funding for sub-acute services found that most tools focussed on either “assistance” (12 tools) or “difficulty or problem” (12 tools); two tools addressed both perspectives [22]. “Difficulty” is the concept underlying the ICF qualifiers of Activities and Participation. Difficulty with activities is widely used in surveys as a self-report measure of disability. The concept of difficulty underpinned global disability estimates in the World Report on Disability [4]. The WHODAS 2.0 provides a generic instrument to assess difficulty on ICF Activities and Participation domains [16].

Both searches carried out for the two Australian national programs described in this paper sought instruments to measure the need for assistance or support, as the measurement construct most relevant for the policy purposes of the programs. For the NDIS, the concept of “assistance” in each life area is of key importance, since the Scheme focuses on supports needed to achieve goals. Personal assistance is a form of support well recognised in the disability field and in population surveys in Australia, as evidenced by its presence in national data collections relating to disability and to disability services [51,52]. A simple “need for assistance” measure has been used in the ABS Survey of Disability, Ageing and Carers as a self-report question (about frequency of support needed), and also successfully used in the national data collection from disability service providers [48,51]; this could be a useful construct in future Australian applications, for continuity of statistical series among other reasons. Learning from and building on the existing measures of functioning (see e.g., Supplementary Tables) will enhance the development process in terms of both efficiency and quality.

#### 4.3.4. Environmental Factors and the Interactions

An IMF must take account of the person’s environment [6]. Indeed, most national programs relevant to functioning, disability and health seek to influence the environment, generally to enhance participation and health and to minimise the risk of disability or disease. The measure should provide a way of recording interactions between environmental factors and functioning domains. This would enable functioning outcomes to be indicated by a change in the environmental interventions needed. 

There are at least two possible approaches to incorporating ICF environmental factor domains in a measurement instrument. The first is to record environmental facilitators or barriers as they affect overall functioning, and the second is to record the facilitators and barriers for each domain of functioning (e.g., mobility, interpersonal relationships) [6] (Annex 2). The first approach is mentioned by Kostanjsek and colleagues [49]: “It is conceivable … to create a list of environmental factors generically relevant across health conditions that could be added to the list of functioning properties if desired.” The latter approach is taken in the recent development of the YIPE (“Your ideas about participation and environment”), which is an instrument that enables self-report of participation and related environmental factors, using all ICF chapters for each component [47]. The tests of YIPE are enabling exploration of the concept of “satisfaction” with participation and its relationship to environmental factors in the person’s life; the YIPE is based on the Australian national data standards for functioning and disability, based in turn on the ICF; early testing of the YIPE may illuminate methods for operationalising this ICF option. (The Australian data standards provide two qualifiers each for Activities and Participation, as a means of distinguishing these two concepts, which have separate definitions but share a single list of life domains. The data standards use the possibility offered in the ICF, of developing additional qualifiers ‘such as a qualifier for involvement or subjective satisfaction’ [6] (p. 231). The Australian “satisfaction with participation” qualifier is a summary indicator reflecting the view of the person about his or her own “involvement in life situations”, and incorporates ideas such as choice, control, importance, and the sense of inclusion. It was developed and subjected to consultation and brief testing during the development of the ICF and the national data standards (late 1990s and early 2000s)). 

#### 4.3.5. Flexibility and Variability

An IMF based on the ICF could offer some flexibility about selection (of both domains and measures) and guidance on criteria and processes. It could offer the benefit of being able to “drill down” to take advantage of the depth of ICF, for example to explore additional detail in areas requiring intensive support (where the added information merits any extra time taken). The construction of the IMF could allow subsets of domains and measurement constructs to be meaningfully selected for instruments focused on specific purposes, populations or health conditions. Such options can be enabled by computer adaptive testing, with guidance on the basis for selections to promote both efficiency and relevance. Other forms of flexibility may require exploration, to ensure relevance to different age groups and varying cultures.

Cultural variation within and across populations requires thought when measuring functioning in national programs. In Australia, for example, around 28% of the population in 2013 was born overseas [53] and nearly one in five people spoke a language other than English at home [54]. Australian Aboriginal and Torres Strait Islander peoples, as the colonised peoples of the land, require particular attention in the development and application of measurement tools, including awareness of potentially differing and mixed cultural interpretations of functioning, and an examination of cross-cultural applicability of measures. There is limited research, especially driven by Indigenous researchers, on how Indigenous peoples define and conceptualise “health”, “participation” and “functioning”. Too often, assessment and screening tools for functioning are not tested for validity and applicability for Indigenous peoples [55,56]. Gilroy and colleagues [57,58] suggest that the approach to empowering Indigenous peoples, in developing and applying a measuring tool, is to explore how to support Indigenous and non-Indigenous peoples to bridge the cultural interface. If an IMF is to be truly applicable across cultures, development and testing should not be mono-cultural. That cross-cultural design can be effective is demonstrated by the collaborative development of a flexible monitoring tool for community-based rehabilitation (CBR) incorporating the ICF [59]. For an instrument to be responsive to cultural diversity, even within a western nation like Australia, peoples of a range of cultural backgrounds must be involved in the planning, development and testing process. 

### 4.4. The ICF, Environmental Factors and Public Health

The ICF and the discussion in this paper have a strong resonance with some fundamental ideas and approaches in public (or population) health [60], for example:

- consideration of diverse populations, in the context of public health efforts aimed at improving the level and equity of health across populations;
- functioning as a health “outcome” in the context of the prevention and promotion functions of public health;
- integration of services (including across sectors) as a means of promoting broad public health objectives, embodied in social determinants and “health in all policies” approaches to public health [61].

According to some authors, there has been a change from “regarding disability as the failed outcome of public health prevention to recognising disability as part of the natural continuum of human experience” [61,62] (p. 14, citing [63]). They note the importance of being able to monitor the health of people with disability, describing disability as a “risk factor” or “determinant” of health and a “stratifying variable” in analysis. This in turn requires the ability to identify people with disability in the population—in the terms of the present paper, to define one or more thresholds, so as to create a subgroup of people on this “natural continuum”. But this is not the only way in which concepts of public health and disability can usefully be harmonised. “Prevention” in public health should not be discussed just as prevention of disease, including prevention of diseases likely to be associated with ongoing disability. It could also embrace the ICF model illustrating the environmental factors which tend to increase disability; that is, it could consider the environmental risks for disability alongside risks for disease. The discussion of “healthy environments” in a public health context could adopt the approach of the ICF, describing the environment in neutral language and recognising that many “factors” can be either barriers (risks) to, or facilitators of health and functioning (e.g., water, air, attitudes, policies). Such a unified approach to analysing and measuring environmental factors would support a unified epidemiology of health and disability.

## 5. Conclusions 

This paper identifies a major gap in the assessment of functioning and disability. It does so by analysing in-depth content reviews of the main available instruments and their relevance to existing policies in Australia. To our knowledge, this is the first paper to fully document this gap in relation to measurement needs for policy implementation in rehabilitation, disability support, and related fields.

The findings of these two Australian searches—and their similarity—are relevant in policy development and information management internationally. An integrative measure of functioning (IMF) would support integrative and person-centred services, and serve the needs of national programs in disability and rehabilitation for generic measures and harmonised language. In public health, there is a need to describe the health status of populations and identify policies that affect overall health. Unified measurement of health status (not just disease status) across all domains of life is required, with a more comprehensive and unified approach to understanding environmental influences. The ICF concepts of functioning and environmental factors would form the basis of an IMF and contribute to the development of public health concepts.

This paper outlines the potential development of an IMF in terms of the framing of policy and service purposes, of information requirements serving these purposes, and of measurement concepts relevant to the information required. Working towards a measure based on the ICF Activities and Participation chapters, incorporating environmental factors and including measures of “need for support or assistance” would be an ideal starting point and would provide a partner instrument to the WHODAS which uses “difficulty” as its measure. Such an IMF could deliver a range of benefits, including supporting better care by providing comprehensive information on functioning across all life domains, facilitating data sharing and communication across service interfaces to promote continuity of care, and reducing the burden and cost associated with repeated assessment. It could also provide a basis for harmonising the conceptual approach to and measurement of functioning in fields such as chronic disease, aged care and public health where successful functioning is a core aim of the service system.

The identification of the need for a generic instrument for measuring functioning, able to be used for individual assessment and monitoring for large national programs in the health and disability fields, is particularly important because some available instruments may appear relevant but are not adequate. Planners and policy makers may be insufficiently aware of this gap and the inefficiencies and lack of comparable data resulting from a plethora of different instruments. Identifying this gap and outlining methods and requirements for its solution are important first steps. Undertaking the development and testing of a new instrument to fill this gap is then needed. This research and development will be informed by the methods, guidance and criteria outlined in this paper. 

## Supplementary Files

Supplementary File 1

## Legislation

National Disability Insurance Scheme Act 2013 (Commonwealth of Australia). Last viewed 23 December 2014: http://www.comlaw.gov.au/Details/C2013A00020.
