# Supplementary material for: In Search of an Integrative Measure of Functioning
Source: Int J Environ Res Public Health. 2015 May 26;12(6):5815–32. doi: 10.3390/ijerph120605815 (PMC4483673; doi:10.3390/ijerph120605815)
Supplement: Supplementary File 1 [file ijerph-12-05815-s001.pdf]

Supplementary Information

In Search of an Integrative Measure of Functioning

Supplementary Tables S1 and S2

Analysis of Instruments Considered for Australian National Applications.

This Appendix outlines information from two separate review processes [21,22,33] that were undertaken to identify measures of functioning that are potentially relevant to two major national programs in Australia.

Table S1 presents information from the evaluation of existing needs assessment tools for possible use in the National Disability Insurance Scheme (NDIS)—tools that can be used to assess the support needs of people with disability and to inform decisions as to resource allocation to meet those needs. Instruments were sourced by several means including advice from eminent researchers and practitioners in various fields of disability (n = 15), consultation with 10 disability and/or carer advocacy organisations, and literature searches. Twenty-nine potential instruments were identified and this list was reduced by application of the following exclusionary criteria:

- open-ended question instruments where the responses did not result in a numerical scale that could be statistically manipulated;
- instruments that contained rating scales but lacked published evidence of basic psychometric properties;
- instruments that contained rating scales with published evidence as to some desirable psychometric properties but had significant limitations with reference to people with disability in an insurance scheme, e.g., significant ceiling effects, limited sensitivity to change.

Finally, instruments were excluded that were inconsistent with key requirements of the Scheme. This process led to 13 instruments being considered against key selection criteria, under the headings of applicability for other Scheme purposes, psychometric properties, ease of use, training needed and cost. Examples of instruments reviewed are provided in Table S1. Not all instruments reviewed are presented as some were provided when under development and as commercial-in-confidence and each of the mental health instruments (LSP-39, CAN and HoNOS) [64–68] had similar ICF coverage or lack of coverage as the BASIS-32.

Table S2 summarises the evaluation of functional assessment instruments in relation to their suitability for classifying sub-acute episodes of care, particularly in order to identify instruments capable of explaining resource use and able to address deficiencies in existing tools [22,33]. Instruments were identified by literature searches and expert recommendations. The National Centre for Classification in Health conducted an initial review to select instruments for further consideration. A survey containing the resulting list of instruments was circulated to a wide range of stakeholders, seeking comments on the instruments and suggestions of additional instruments. Seventy-four organisations responded (102 individual responses were received from within these organisations).

Over 50 instruments were considered in the report [22]. Some were excluded without detailed evaluation as they were clearly unrelated to the measurement purposes required.

Thirty-three instruments were selected to be linked to the ICF, using standardised linking rules [18]. In addition to coverage of the ICF domains, the "perspective" and "response options" were tabulated, e.g., "dependency" (need for assistance) and "extent of functioning problem" (difficulty). Other criteria applied in the review of instruments included published evidence of instrument validity and reliability, clinical utility, and cost and licensing arrangements. To be suitable for activity-based funding an instrument should be clinician-rated and should measure the need for assistance. Table S2 does not include 9 of the 33 instruments linked to ICF: 5 instruments specific to palliative care; 2 instruments that did not map to any ICF Activities and Participation (A&P) domains; one instrument targeted at children for a specific (orthopaedic) purpose; and one instrument specific to 'dependency' assessment in residential aged care in Australia.

Table S1. Selected instruments reviewed for the National Disability Insurance Scheme.

Instrument	Extent of ICF Coverage (A&P)	Measurement Construct(s)	Age Group Restrictions/Focus	Commentary
**AusTOMs** [69,70]	Comprehensive<br>The OT AusTOMs cover all areas except Communication. This is covered in detail in the SP AusTOMs. The PT scales focus more strongly on Body Functions.	Extent of impairment (none to severe/complete)<br>Activity limitation present (none to profound/complete) or activity limitation (degree of difficulty)	Adults and children	A well validated instrument. Its focus on therapy disciplines, rather than the person, introduces some complexities but otherwise it is a simple, informative and sensitive instrument. Measures difficulty; modifications would be required for it to perform as a measure of support needs.
**BASIS-32** [71]	Limited<br>There are elements addressing Learning and Applying Knowledge, General Tasks and Demands, Domestic Life, Interpersonal Interactions and Relationships and Major Life Areas.<br>Communication, Mobility, Self-Care are not addressed.	Degree of difficulty	Adolescents and adults	This is a robust instrument for the measurement of psychopathology, mental wellbeing and function with people with serious mental illness. The domains do not cover the needs of people with physical disability.
**Inventory for client and agency planning (ICAP)** [72]	Moderate<br>Pre-dates the ICF and is therefore not constructed to the ICF domains. However, coverage of activity and participation is broad, with elements addressing all A&P domains other than Major Life Areas.	Abilities (adaptive behaviour) and how well the activity is completed without help<br>Behaviours (frequency and severity of behaviours of concern)	Adults and children	As an older instrument some of the language and examples need updating. The instrument would benefit from being shortened. The compelling feature of this instrument is its high level of support variance explanation reported in the DOORS system in the United States.
**Impact on Participation and Autonomy (IPA)** [73]	Moderate<br>Mobility, Self-Care, Domestic Life, Interpersonal Interactions and Relationships are addressed as are some Major Life Areas. Learning and Applying Knowledge, General Tasks and Demands and Communication are not specifically addressed.	People's views (self-rating) of the likelihood (chances of …) certain outcomes (e.g. fulfilling my role at home as I would like). Five point scale from zero (very good) to four (very poor)	Adolescents and adults	An interesting and useful instrument with great potential in disability support services; focuses very much on people's views as to their participation and the extent to which this is a problem/not a problem.<br>The response selected represents the result of activity/participation (limitations/restrictions) and the interaction with the environment (facilitators and barriers). As such it is not a primary measure of support needs.

Table S1. *Cont.*

Instrument	Extent of ICF Coverage (A&P)	Measurement Construct(s)	Age Group Restrictions/Focus	Commentary
**Supports Intensity Scale (SIS)** [74]	Limited<br>Not constructed to an obvious ICF relationship. Mobility is not well addressed. There is some coverage of Learning and Applying Knowledge, General Tasks and Demands, Communication, Self-Care, Domestic Life. Interpersonal Interactions and Relationships are more strongly addressed as are Major Life Areas.	Frequency of activity<br>Daily time required<br>Type of support (none to full physical assistance)	Adults with intellectual disability	Given the limited coverage of ICF, a lack of sensitivity with people with physical disability was anticipated and was reported in the literature.
**WHODAS 2.0 (36 item)** [75]	Moderate<br>Constructed to the ICF and covers Mobility, Self-Care, Domestic Life, Interpersonal Interactions and Relationships and some Major Life Areas.<br>Combines Learning and Applying Knowledge, General Tasks and Demands and Communication as Cognition. Coverage of communication is therefore limited.	Degree of difficulty (none to extreme/cannot do)	Adults	Measures difficulty. There are risks of ceiling effects with people with significant disability[3]. Insensitivity to the mobility capacity of wheelchair users (item 2.5 requires people to respond to an item "walking a long distance …")<br>*See also Table S1.*

Table S2. Instruments reviewed for application to activity based funding for sub-acute services.

Instrument	ICF Components Included	Perspective (P) & Response Options (R)	Age Group Focus of Instrument	Commentary
Activities of Daily Living Questionnaire (ADLQ) [76]	d1, d3, d4, d5, d6, d8, d9	P: Extent of functioning problem<br>R: Intensity	Adults: elderly, community living, broad functional skills<br>Assessment of functional abilities, in patients with probable Alzheimer disease and other forms of dementia	Could be a useful scale to assess functioning amongst older patients with dementia.

Table S2. *Cont.*

Instrument	ICF Components Included	Perspective (P) & Response Options (R)	Age Group<br>Focus of Instrument	Commentary
Assessment of Living Skills and Resources (ALSAR) [77,78]	d1, d3, d4, d5, d6, d8, d9<br>e1, e5	P: Dependency<br>R: Intensity	Adults—instrumental activities of daily living<br>Instrumental activities of daily living (IADL)	Good coverage of ICF A&P domains. Has good psychometric properties. Administration is complex and demands more time than other instruments reviewed. It can be used to assess maintenance of independent life in the community, identify needs, assess risk, prioritise treatment goals and promote interdisciplinary problem solving. Designed for adults over 65 living in the community, but could potentially be used for adults generally in community settings.
Australian Therapy Outcome Measures (AusTOMs) [69,70]	b1<br>d1, d2, d4, d5, d6, d7, d8, d9	P: Extent of functioning problem / dependency<br>R: Intensity	Adults, child & youth<br>Changes in clients' Impairments, Activity (Limitations) and Participation (Restrictions), Wellbeing/Distress	AusTOMs measure difficulty, not need for assistance, and are a set of distinct domains, with no metric specified to combine domain scores. The concurrent validity and inter-rater reliability have been demonstrated; no studies that have assessed the predictive validity of the tool.
Bristol Activities of Daily Living Scale (BADLS) [79]	b1, b5, b6<br>d3, d4, d5, d6, d8, d9<br>e1	P: Extent of functioning problem<br>R: Intensity	Adults: elderly, community living, broad functional skills<br>Ability to perform activities of daily living	Although covering a range of ICF domains well, was excluded as it is designed to be completed via the observations of a relative or friend over a two-week period.
Barthel Index (BI) [80]	b5, b6<br>d4, d5<br>e3	P: Dependency<br>R: Intensity	Adults—rehabilitation settings<br>Level of assistance required to perform activities of daily living	Very widely used with older people; however, it has important coverage limitations and is therefore frequently used alongside other instruments.
Clinical Dementia Rating (CDR) [81,82]	b1<br>d1, d5, d6, d7, d8, d9	P: Extent of functioning problem<br>R: Presence of problem / frequency / intensity	Adults—psycho-geriatric<br>Diagnostic and staging scale for Dementia	Reports on the clinical stage of dementia. Some coverage of ICF A&P domains is provided.
Frenchay Activities Index (FAI) [83]	d1, d4, d6, d8, d9	P: Extent of functioning problem<br>R: Frequency	Adults: elderly, community living, broad functional skills<br>Measure of instrumental activities of daily living	An outcome scale with limited coverage of ICF domains.

Table S2. *Cont.*

Instrument	ICF Components Included	Perspective (P) & Response Options (R)	Age Group<br>Focus of Instrument	Commentary
Functional Disability Inventory (FDI) [84]	b1<br>d1, d4, d5, d6, d8, d9	P: Extent of functioning problem<br>R: Intensity	Child & youth<br>Degree to which children experience difficulty in physical and psychosocial functioning due to their physical health status	Judged unsuitable as the items are primarily restricted to those activities most likely to be affected by chronic pain.
Functional Independence Measure (FIM) [30,85,86]	b1, b5, b6<br>d1, d2, d3, d4, d5, d6, d7<br>e1, e3	P: Dependency<br>R: Intensity	Adults—rehabilitation settings<br>Level of assistance required to perform activities of daily living	Widely used, psychometric properties well tested. There was clinician support for the FIM for rehabilitation inpatients.<br>In survey results, limitations particularly noted were: ceiling and floor effects; lack of validity and/or limited sensitivity for certain groups, in particular, people with psychogeriatric disorders, ambulatory/outpatients and people living in the community; training costs.<br>Inadequate overage of ICF chapters 6 to 9.
Health of the Nation Outcome Score (HoNOS) [32,87]	b1<br>d5, d7<br>e1, e3, e4	P: Extent of functioning problem<br>R: Intensity	Adults—psycho-geriatric<br>Measure consumer outcomes in inpatient and ambulatory mental health services	Psychometric properties generally positive. Use designed for mental illness and fortnightly monitoring by clinicians and therefore resource demanding. Focus on difficulty. Applicability for ABF is likely to be low.
Lawton's Instrumental Activities in Daily Living (IADL) [88]	d2, d3, d4, d5, d6, d8	P: Dependency<br>R: Intensity	Adults—instrumental activities of daily living<br>Measure of instrumental activities of daily living	Used in the assessment of people's performance of instrumental activities of daily living; less comprehensive than more modern instruments.
Inventory for Client and Agency Planning (ICAP) [72]	b1, b2, b5, b6<br>d1, d2, d3, d4, d5, d6, d7, d8, d9<br>e1, e3, e5	P: Extent of functioning problem<br>R: Intensity	Adults: people with disabilities (covered under national disability agreement)<br>Level of support required based on: a) level of functioning, and b) the presence/absence of maladaptive behaviours	Did not meet requirements of being short and easy to complete and/or having clinical utility.
Impact on Participation and Autonomy Questionnaire (IPA) [73]	d3, d4, d5, d6, d7, d8, d9<br>e3	P: Self-determination<br>R: Intensity	Adult—general<br>Assess participation and autonomy	Did not meet requirements of being short and easy to complete and/or having clinical utility.

Table S2. *Cont.*

Instrument	ICF Components Included	Perspective (P) & Response Options (R)	Age Group<br>Focus of Instrument	Commentary
Katz Index of Activities of Daily Living (KIADL) [81]	b5, b6<br>d4, d5<br>e1	P: Dependency<br>R: Intensity	Adults: elderly, community living, broad functional skills<br>Ability to perform activities of daily living independently	The six domains in the KIADL are covered within the FIM and would therefore be redundant when the FIM is used. The FIM is preferred over the KIADL for reasons of its demonstrated statistical power.
Leeds Assessment and Scale of Handicap (LASH) [81]	b1<br>d4, d9<br>e3	P: Extent of functioning problem/dependency<br>R: Intensity	Adults—rehabilitation settings<br>Difficulties in mobility, physical independence, orientation and social integration	Designed for people with brain injuries in inpatient rehabilitation settings. The four domains do not map well to the ICF.
London Handicap Scale (LHS) [89]	d4, d7, d8, d9	P: Extent of functioning problem<br>R: Intensity	Adult—general<br>Impact of a health condition on functioning	*No commentary provided*
Multidimensional Assessment of Neurodegenerative Symptoms (MANS) [90]	b1, b6, b7<br>d1, d2, d3, d4, d6, d7, d8	P: Extent of functioning problem<br>R: Presence of problem/frequency	Adults—psycho-geriatric<br>Changes in daily habits, personality and motor functioning	Relevant to neurodegenerative aetiologies.
Northwick Park Dependency Scale (NPDS) [81]	b1, b5, b6, b8<br>d3, d4, d5, d6<br>e1, e3	P: Dependency<br>R: Intensity/frequency/ statements	Adults—rehabilitation settings<br>Quantify an individual's needs for nursing care and support	Designed to assess the nursing dependency of people with neurological conditions (principally used with traumatic brain injury and stroke).
Participation Objective Participation Subjective (POPS) [81]	d2, d4, d6, d7, d8, d9	P: Information/satisfaction<br>R: Intensity	Adults, child & youth<br>Address participation	Combination of self-report ("subjective") and assessor reporting ("objective"); scoring system for the objective component is "complex and involved, and the need for a statistical program to calculate scores may limit the feasibility of using the POPS in a clinical setting"
Rivermead Activities of Daily Living (RADL) [81]	d4, d5, d6, d8<br>e3	P: Dependency<br>R: Statements	Adults: elderly, community living, broad functional skills<br>Measure instrumental activities of daily living	Has a number of strengths but the range of ICF domains covered is limited.
Resource Utilisation Groups – Activities of Daily Living (RUG-ADL) [91,92]	d4, d5<br>e3	P: Dependency<br>R: Intensity	Palliative care (& Adults: resource allocation)<br>Classification of long-term care residents	Floor and ceiling effects and a lack of sensitivity were described as being limitations. Inadequate overage of ICF chapters 6 to 9. Does not cover psychosocial and spiritual domains.

Table S2. *Cont.*

Instrument	ICF Components Included	Perspective (P) & Response Options (R)	Age Group<br>Focus of Instrument	Commentary
Functional Autonomy Measurement System (SMAF) [81]	b1, b2, b5, b6<br>d3, d4, d5, d6, d8<br>e1, e3	P: Dependency<br>R: Intensity	Adults: elderly, community living, broad functional skills<br>Disabilities and whether available physical and social resources are adequate	Provides coverage of some Environmental Factors, and useful in planning for, and monitoring, community living. The SMAF is likely to be too long to be acceptable.
Functional Independence Measure for Children (weeFIM) [93]	b1, b5, b6<br>d1, d3, d4, d5, d9<br>e3	P: Dependency<br>R: Intensity	Child & youth<br>Level of assistance required in self-care, mobility, and cognition	Age limitation (designed for ages 6 months to 7 years). For other comments see FIM.
WHODAS 2.0 [75]	b1<br>d1, d2, d3, d4, d5, d6, d7, d8, d9<br>e1, e3, e4	P: Extent of functioning problem/impact<br>R: Intensity/frequency (impact)	Adult—general<br>Difficulties due to health conditions	Measures difficulty. The construct and discriminant validity and test-retest reliability have been established; no reported studies assessing the predictive validity. Some items may be difficult to judge in an inpatient environment. Testing of the reliability of the instrument as an observer-completed instrument remains to be conducted. While freely available, modification would require agreement from WHO.

Notes: A selection of references used in the source reports [21,22] is included here, to identify the instruments. This list does not include all of the wider range of references used to reach conclusions about the instruments' suitability.

References

64. Rosen, A.; Hadzi-Pavlovic, D.; Parker, G.; Trauer, T. The life skills profile: Background items and scoring for the LSP-39, LSP-20 and LSP-16, 2006. Available online: http://www.blackdoginstitute.org.au/docs/LifeSkillsProfile.pdf (accessed on 28 January 2012).
65. Trauer, T.; Tobias, G. The camberwell assessment of need and behaviour symptom identification scale as routine outcome measures in psychiatric disability rehabilitation and support service. *Commun. Ment. Health J.* **2004**, *40*, 211–221.
66. Trauer, T.; Tobias, G.; Slade, M. Development and Evaluation of a Patient-rated Version Of the Camberwell Assessment of Need Short Appraisal Schedule (CANSAS-P). *Commun. Ment. Health J.* **2007**, *44*, 113–124.
67. Brann, P.; Coleman, G. On the meaning of change in a clinician's routine measure of outcome: HonOSCA. *Aust. N. Zeal. J. Psychia.* **2010**, *44*, 1097–1104.
68. Fleminger, S.; Leigh, E.; Eames, P.; Langrell, L.; Logsdail, S. HoNOS-ABI: A reliable outcome measure of neuropsychiatric sequelae to brain injury? *Psychiatr. Bull.* **2005**, *29*, 53–55.
69. Australian Therapy Outcome Measures (AusTOMs). Available online: http://www.latrobe.edu.au/austoms/ (accessed on 24 May 2007).
70. Perry, A.; Morris, M.; Unsworth, C.; Duckett, S.; Skeat, J.; Dodd, K.; Taylor, N.; Reilly, K. Therapy outcome measures for allied health practitioners in Australia: The AusTOMs. *Int. J. Qual. Health Care* **2004**, ***16***, 285–291.
71. Eisen, S.V.; Wilcox, M.; Leff, H.S.; Schaefer, E.; Culhane, M.A. Assessing behavioral health outcomes in outpatient programs: Reliability and validity of the BASIS-32. *J. Behav. Health Serv. Res.* **1999**, *26*, 5–17.
72. Bruininks, R.H.; Hill, B.K.; Weatherman, R.F.; Woodcock, R.W. *Examiner's Manual. ICAP: Inventory for Client and Agency Planning*; DLM Teaching Resources: Allen, TX, USA, 1986.
73. Cardol, M.; de Haan, R.J.; de Jong, B.A.; van den Bos, G.A.M.; de Groot, I.J.M. Psychometric properties of the Impact on Participation and Autonomy Questionnaire. *Arch. Phys. Med. Rehabil.* **2001**, *82*, 210–216.
74. Smit, W.; Sabbe, B.; Prinzie, P. Reliability and validity of the supports intensity scale (SIS) measured in adults with physical disabilities. *J. Dev. Phys. Disabil.* **2011**, *23*, 277–287.
75. Ustun, T.B.; Chatterji, S.; Kostanjsek, N.; Rehm, J.; Kennedy, C.; Epping-Jordan, J.; Saxena, S.; von Korff, M.; Pull, C. Developing the World Health Organization disability assessment schedule 2.0. *Bull. WHO* **2010**, *88*, 815–823.
76. Johnson, N.; Barion, A.; Rademaker, A.; Rehkemper, G.; Weintraub, S. The activities of daily living questionnaire - A validation study in patients with dementia. *Alzheimer Dis. Assoc. Disord.* **2004**, *18*, 223–230.

77. Drinka, T.; Williams, J.; Schram, M.; Farrell-Holtan, J.; Euhardy, M. *Assessment of Living Skills and Resources (ALSAR): An Instrumental Activities of Daily Living Assessment, in Comprehensive Geriatric Assessment;* Osterweil, D., Brummel-Smith, K., Beck, J., Eds.; McGraw-Hill: New York, NY, USA, 2000.
78. Clemson, L.; Bundy, A.; Unsworth, C.; Singh, M.F. Validation of the modified assessment of living skills and resources, an IADL measure for older people. *Disabil. Rehabil.* **2009**, *31*, 359–359.
79. Bucks, R.S.; Ashworth, D.L.; Wilcock, G.K.; Siegfried, K. Assessment of activities of daily living in dementia: Development of the Bristol activities of daily living scale. *Age Ageing* **1996**, *25*, 113–120.
80. Van der Putten, J.J.; Hobart, J.C.; Freeman, J.A.; Thompson, A.J. Measuring change in disability after inpatient rehabilitation: Comparison of the responsiveness of the Barthel index and the functional independence measure. *J. Neurol. Neurosurg. Psychiat.* **1999**, *66*, 480–484.
81. Tate, R. *A Compendium of Tests, Scales and Questionnaires: The Practitioner's Guide to Measuring Outcomes after Acquired Brain Impairment*; Psychology Press: New York, NY, USA, 2010.
82. Hirdes, J.P.; Ljunggren, G.; Morris, J.N.; Frijters, D.H.M.; Soveri, H.F.; Gray, F.; Björkgren, F.; Gilgen, R. Reliability of the interRAI suite of assessment instruments: A 12-country study of an integrated health information system. *BMC Health Serv. Res.* **2008**, *8*, 277–277.
83. Wade, D.T.; Legh-Smith, J.; Hewer, R.L. Social activities after stroke: measurement and natural history using the Frenchay Activities Index. *Int. Rehabil. Med.* **1985**, *7*, 176–181.
84. Kashikar-Zuck, S.; Flowers, S.R.; Claar, R.L.; Guite, J.W.; Logan, D.E.; Lynch-Jordan, A.M.; Palermo, T.M.; Wilson, A.C. Clinical utility and validity of the functional disability inventory among a multicenter sample of youth with chronic pain. *Pain* **2011**, *152*, 1600–1607.
85. Ottenbacher, K.J.; Hsu, Y.; Granger, C.V.; Fiedler, R.C. The reliability of the functional independence measure: a quantitative review. *Arch. Phys. Med. Rehabil.* **1996**, *77*, 1226–1232.
86. Stineman, M.G.; Shea, J.A.; Jette, A.; Tassoni, C.J.; Ottenbacher, K.J.; Fiedler, R.; Granger, C.V. The functional independence Measure: Tests of scaling assumptions, structure, and reliability across 20 diverse impairment categories * 1,* 2,* 3,* 4. *Arch. Phys. Med. Rehabil.* **1996**, *77*, 1101–1108.
87. Coetzer, R. HoNOS—ABI: A clinically useful outcome measure? *Psychiatr. Bull.* **2001**, *25*, 421–422.
88. Lawton, M.P.; Brody, E.M. Assessment of older people: self-maintaining and instrumental activities of daily living. *Gerontology* **1969**, *9*, 179–186.
89. Harwood, R.H.; Rogers, A.; Dickinson, E.; Ebrahim, S. Measuring handicap: The London Handicap Scale, a new outcome measure for chronic disease. *Qual. Health Care* **1994**, *3*, 11–16.
90. Locke, D.E.; Dassel, K.B.; Hall, G.; Baxter, L.C.; Woodruff, B.K.; Hoffman Snyder, C.; Miller, B.L.; Caselli, R.J. Assessment of patient and caregiver experiences of dementia-related symptoms: Development of the multidimensional assessment of neurodegenerative symptoms questionnaire. *Dement. Geriatr. Cogn. Disord.* **2009**, *27*, 260–272.
91. Waller, A.; Girgis, A.; Lecathelinais, C.; Scott, W.; Foot, L.; Sibbritt, D.; Currow, D.; Palliative Care Research Program Team. Validity, reliability and clinical feasibility of a needs assessment tool for people with progressive cancer. *Psycho. Oncology* **2010**, *19*, 726–733.
92. Australian Health Services Research Institute. Palliative Care Outcomes Collaboration: RUG-ADL & AKP Assessment. Available online: http://www.pcoc.org.au/ (accessed on 10 October 2012).

93. Msall, M.E.; DiGaudio, K.; Rogers, B.T.; LaForest, S.; Catanzaro, N.L.; Campbell, J.; Wilczenski, F.; Duffy, L.C. The functional independence measure for children (WeeFIM): Conceptual basis and pilot use in children with developmental disabilities. *Clin. Pediatr.* **1994**, *33*, 421–430.

© 2015 by the authors; licensee MDPI, Basel, Switzerland. This article is an open access article distributed under the terms and conditions of the Creative Commons Attribution license (http://creativecommons.org/licenses/by/4.0/).
